# Disease specific and nonspecific metabolic brain networks in behavioral variant of frontotemporal dementia

**DOI:** 10.1002/hbm.26140

**Published:** 2022-11-05

**Authors:** Tomaž Rus, Matej Perovnik, An Vo, Nha Nguyen, Chris Tang, Jan Jamšek, Katarina Šurlan Popović, Timo Grimmer, Igor Yakushev, Janine Diehl‐Schmid, David Eidelberg, Maja Trošt

**Affiliations:** ^1^ Department of Neurology UMC Ljubljana Ljubljana Slovenia; ^2^ Medical Faculty University of Ljubljana Ljubljana Slovenia; ^3^ Center for Neurosciences Feinstein Institutes for Medical Research Manhasset New York USA; ^4^ Department of Genetics Albert Einstein College of Medicine Bronx New York USA; ^5^ Department of Nuclear Medicine UMC Ljubljana Ljubljana Slovenia; ^6^ Department of Neuroradiology, Clinical Institute of Radiology UMC Ljubljana Ljubljana Slovenia; ^7^ Department of Psychiatry and Psychotherapy, Klinikum rechts der Isar Technical University of Munich, School of Medicine Munich Germany; ^8^ Department of Nuclear Medicine, Klinikum rechts der Isar Technical University of Munich Munich Germany; ^9^ TUM Neuroimaging Center, Klinikum rechts der Isar Technical University of Munich Munich Germany

**Keywords:** behavioral variant of frontotemporal dementia, default mode network, FDG‐PET, functional connectivity, network analysis, SSM/PCA

## Abstract

Behavioral variant of frontotemporal dementia (bvFTD) is common among young‐onset dementia patients. While bvFTD‐specific multivariate metabolic brain pattern (bFDRP) has been identified previously, little is known about its temporal evolution, internal structure, effect of atrophy, and its relationship with nonspecific resting‐state networks such as default mode network (DMN). In this multicenter study, we explored FDG‐PET brain scans of 111 bvFTD, 26 Alzheimer's disease, 16 Creutzfeldt‐Jakob's disease, 24 semantic variant primary progressive aphasia (PPA), 18 nonfluent variant PPA and 77 healthy control subjects (HC) from Slovenia, USA, and Germany. bFDRP was identified in a cohort of 20 bvFTD patients and age‐matched HC using scaled subprofile model/principle component analysis and validated in three independent cohorts. It was characterized by hypometabolism in frontal cortex, insula, anterior/middle cingulate, caudate, thalamus, and temporal poles. Its expression in bvFTD patients was significantly higher compared to HC and other dementia syndromes (*p* < .0004), correlated with cognitive decline (*p* = .0001), and increased over time in longitudinal cohort (*p* = .0007). Analysis of internal network organization by graph‐theory methods revealed prominent network disruption in bvFTD patients. We have further found a specific atrophy‐related pattern grossly corresponding to bFDRP; however, its contribution to the metabolic pattern was minimal. Finally, despite the overlap between bFDRP and FDG‐PET‐derived DMN, we demonstrated a predominant role of the specific bFDRP. Taken together, we validated the bFDRP network as a diagnostic/prognostic biomarker specific for bvFTD, provided a unique insight into its highly reproducible internal structure, and proved that bFDRP is unaffected by structural atrophy and independent of normal resting state networks loss.

## Graphical abstract

Behavioral variant of the frontotemporal dementia (bvFTD) specific metabolic brain pattern (bFDRP) is a robust metabolic biomarker validated on three different populations. Its internal structure is characterized by prominent network disruption and inefficient information transfer in bvFTD patients. Despite overlapping with the default mode network, bFDRP is an independent network with highly reproducible changes.

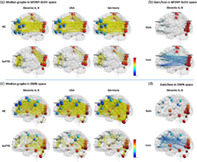

## 1 INTRODUCTION

Frontotemporal dementia (FTD) is the third most common cause of neurodegenerative dementia after Alzheimer's disease (AD) and dementia with Lewy bodies (DLB) (Erkkinen et al., 2018). While it accounts for about 2.7% cases (Erkkinen et al., 2018; Hogan et al., 2016), it is an important cause of cognitive decline, especially in young onset cases. In patients below 50 years, it is even more common than AD (Hendriks et al., 2021).

FTD is an umbrella term that includes several clinical syndromes related to diverse neurodegenerative processes. However, all these syndromes are characterized by frontal, temporal, insular, and subcortical dysfunction clinically manifested as a decline in executive function, social cognition, behavior, and language (Erkkinen et al., 2018). The first three domains are predominantly affected in the most common behavioral variant of FTD (bvFTD) (Rascovsky et al., 2011) while different aspects of language impairment are found in somewhat rarer primary progressive aphasias (PPAs) (Gorno‐Tempini et al., 2011). Just as FTD is a clinical umbrella term, frontotemporal lobe degeneration (FTLD) denotes continuum of FTD‐associated pathological changes characterized by the deposition of different proteins in the brain (Mackenzie et al., 2009). The three most common proteins associated with FTLD are tau, TAR DNA‐binding protein 43 (TDP‐43) and RNA‐binding protein FUS, all of them being further subdivided. Due to such diversity not only between different neurodegenerative disorders with similar presentations but also among subtypes, reliable differentiation is important for prognosis, symptomatic treatment and for identifying subjects to be included in disease modification trials (LeWitt et al., 2011; Vanderschaeghe et al., 2017).

Neuroimaging, structural as well as functional, has been proven useful in diagnosis of bvFTD and is included in current diagnostic criteria (Rascovsky et al., 2011). Indeed, visual reading and univariate studies of bvFTD brain metabolism using 2‐[^18^F]fluoro‐2‐deoxy‐d‐glucose (FDG) positron emission tomography (PET) brain scans have shown diagnostic superiority to clinical diagnosis (Foster et al., 2007) with evident progression of metabolic decline in specific brain regions over time (frontal lobes, insula, caudate nuclei, and thalami) (Diehl‐Schmid et al., 2007; Diehl‐Schmid et al., 2014). In addition to univariate studies exploring brain voxels independently, a recent multivariate analysis employing scaled subprofile model/principal component analysis (SSM/PCA) has identified a specific bvFTD‐related pattern (bFDRP) of functionally interconnected brain voxels (Nazem et al., 2018). Multivariate approaches such as SSM/PCA identify discrete brain networks and are especially useful in neurodegenerative research, in which focal brain dysfunction affects the activity of remote areas via neural networks. However, while only a limited number of univariate studies explored relationship between metabolic changes and atrophy in bvFTD (Buhour et al., 2017), this universal finding in bvFTD has not been taken into account in multivariate bFDRP before.

In a given disorder, different brain networks may be involved and their relative contribution may be disease‐specific (Ko et al., 2016; Tang et al., 2010). Many neurodegenerative disorders have therefore been characterized not only by gain of disease‐specific networks but also by changes in normal resting state networks (RSN) such as default mode network (DMN) (Jobson et al., 2021; Schindlbeck et al., 2021). DMN has been rigorously studied in several neurodegenerative disorders using resting state functional MRI (rs‐fMRI). It represents a baseline resting state brain network that deactivates when task/attention‐associated networks are activated (Smallwood et al., 2021), and neurodegenerative processes nonspecifically disrupt it (Grieder et al., 2018). Previous rs‐fMRI studies have shown prominent disruption of nonspecific RSN in bvFTD (Agosta et al., 2013; Day et al., 2013). While the DMN has also recently been identified in an SSM/PCA analysis of FDG PET scans from health subjects (Spetsieris et al., 2015), changes in this network in bvFTD patients have not been studied, nor its relationship with the disease‐specific bvFTDRP topography. Likewise, similarities and differences in functional connectivity have not been rigorously examined within the respective networks. In this multicentric study, we aimed to identify and validate bFDRP in a new independent population, explore its internal structure and the contribution of structural atrophy to the metabolic pattern. Finally, we studied a relationship between disease specific bFDRP and nonspecific DMN.

## 2 METHODS

### 2.1 Subjects

*Cohorts from UMC Ljubljana, Slovenia: Slovenia A (Identification cohort)*: The multivariate metabolic pattern bFDRP‐SLOV was identified in a cohort Slovenia A consisted of 20 patients diagnosed with probable bvFTD according to the current diagnostic criteria (Rascovsky et al., 2011) and 20 age‐matched healthy control subjects (HC) recruited from UMC Ljubljana, Slovenia.

*Slovenia B (Validation cohort)*: The pattern was validated on an independent cohort of another 20 probable bvFTD patients and 20 HC from the same center.

Patients from both cohorts Slovenia A and B underwent brain FDG‐PET as a part of routine clinical evaluation in the period 2013–2022 and were randomly assigned to either of the two cohorts. They underwent clinical and structural neuroimaging evaluation. Individuals with structural brain changes (e.g., ischemic stroke, hemorrhage, brain trauma) were not included. Cerebrospinal fluid (CSF) analysis for Alzheimer's biomarkers (Aβ42, *p*‐tau, *t*‐tau) (Jack et al., 2018) was performed at the Department of Neurology, UMCL, as described previously (Perovnik et al., 2022) in 30 out of 40 bvFTD patients. None of the tested bvFTD patients showed Alzheimer's CSF profile. FDG‐PET imaging was performed on their usual daily medication. Eight patients (5 in Slovenia A and 3 in Slovenia B) were on low doses of atypical neuroleptics; none were receiving dopaminergic therapy, narcotics or corticosteroids at the time of imaging.

*Slovenia C (Other testing cohort)*: To further validate the bFDRP‐SLOV, we analyzed an additional cohort of patients with other neurodegenerative dementia: 26 with dementia due to AD, 16 with sCJD, 8 with svPPA and 6 with nfvPPA. AD patients were all diagnosed clinically with dementia, had Mini‐Mental State Examination score (MMSE) ≤ 25 and fulfilled the NIA‐AA 2018 CSF criteria for AD (Jack et al., 2018). All the sCJD patients had pathologically confirmed diagnosis. Detailed description of AD and sCJD patients is available elsewhere (Rus et al., 2019). PPA patients were diagnosed according to current criteria (Gorno‐Tempini et al., 2011).

*Slovenia D (Longitudinal cohort)*: Longitudinal changes in bFDRP‐SLOV expression were studied in a small cohort of six bvFTD patients that underwent FDG‐PET twice (timespan between the two scans 3.0 ± 2.0 years). Four patients were clinically diagnosed with probable and two with possible bvFTD (Rascovsky et al., 2011). The first scans of two probable bvFTD patients were also part of identification cohort Slovenia A and the other two probable patients of cohort Slovenia B.

### 2.2 Other data sets

The metabolic bFDRP‐SLOV pattern was further validated in two independent cohorts from other centers: cohort *USA* consisted of 25 bvFTD patients and 22 HC that underwent FDG‐PET at Feinstein Institutes for Medical Research, New York, USA, and cohort *Germany*, which consisted of 44 bvFTD, 16 svPPA, 12 nfvPPA patients and 15 HC that underwent FDG‐PET at the Technical University of Munich, Germany. Limited metabolic and clinical data of these subjects have appeared before (Nazem et al., 2018).

### 2.3 FDG‐PET acquisition and processing

Subjects from Slovenian cohorts underwent FDG‐PET brain imaging at the Department of Nuclear Medicine at UMC Ljubljana. After overnight fasting, FDG with 250 MBq (before August 2019; *n*_bvFTD_ = 27, *n*_HC_ = 40) or with 200 MBq activity (after August 2019; *n*_bvFTD_ = 13) was administered followed by rest in a quiet dark room with eyes closed for 30 min. Afterward, low dose attenuation correction CT and FDG‐PET scan was obtained using Siemens Biograph mCT X PET/CT scanner (before August 2019; *n*_bvFTD_ = 27, *n*_HC_ = 40) or Siemens Biograph mCT Flow Edge PET/CT scanner (after August 2019, *n*_bvFTD_ = 13). Images from both scanners were reconstructed to 400 × 400 matrix with voxel size 1.02 × 1.02 × 3 mm and 4 mm Gaussian filter using OSEM + PSF + TOF reconstruction algorithm.

Scanning of validation cohort subjects from USA and Germany was conducted at Feinstein Institute for Medical Research, New York, USA, and at the Technical University of Munich, Germany on local imaging platforms as reported elsewhere (Nazem et al., 2018).

FDG‐PET scans were converted to Analyze format, spatially normalized into Montreal Neurological Institute (MNI)‐based PET template and smoothed using a three‐dimensional Gaussian kernel of 10 × 10 × 10 mm in SPM5 software (Wellcome Trust Centre for Neuroimaging), running in Matlab 7.0 (MathWorks Inc.).

### 2.4 Multivariate SSM‐PCA procedure for metabolic bFDRP‐SLOV identification

Characteristic metabolic brain pattern associated with bvFTD was identified by analyzing bvFTD and HC scans from Slovenia **A** cohort using an automatic voxel‐based SSM/PCA procedure described elsewhere (Spetsieris et al., 2009) (available at http://www.feinsteinneuroscience.org; Center for Neuroscience, Feinstein Institute for Medical Research, NY, USA). Gray matter was extracted using a custom mask based on Automated Anatomical Labeling (AAL) atlas (Rolls et al., 2020) with the addition of pons and midbrain. The SSM/PCA procedure resulted in a set of linearly independent covariance patterns (principal components, PC) and the first six PCs (typically representing approximately 50% of the total subject × voxel variance) were further analyzed. The expression (subject score) of each subject was calculated for each PC and these values entered a series of all possible logistical regression models, with the group (patients vs. controls) as the predicting variable. The optimal model to differentiate between bvFTD and HC subjects was determined according to the lowest Akaike Information Criterion (AIC). Final subject scores were *z*‐scored (normalized) according to HC.

The stability of the pattern was ascertained by the bootstrapping procedure performed using an in‐house MATLAB script (1000 iterations, one‐sided 95% confidence interval (CI)).

### 2.5 Validation and clinical correlations

The bFDRP‐SLOV was validated prospectively on a Slovenia **B**, USA, and Germany subjects. The bFDRP‐SLOV subject scores were calculated in all scans using Topographic Profile Rating algorithm (TPR) (Spetsieris et al., 2009) and *z*‐scored according to Slovenia **A** HC.

Further, we tested the ability of bFDRP‐SLOV to distinguish between bvFTD and other dementia patients. The bFDRP‐SLOV subject scores were calculated in AD, CJD, svPPA, and nfvPPA patients from Slovenia **C** and Germany and compared to the corresponding bvFTD patients from the same site using one‐way ANOVA followed by post‐hoc Bonferroni's comparison.

The bFDRP‐SLOV was then compared to the bFDRP‐NY, which has been previously identified in USA cohort patients (Nazem et al., 2018). The topographical similarity was explored by voxel‐wise Pearson's correlation incorporating a correction for spatial autocorrelation (Ko et al., 2014). Thereafter, we explored the similarity between both patterns subjects scores in individual subjects by calculating and correlating bFDRP‐SLOV and bFDRP‐NY subject scores for bvFTD patients from cohorts Slovenia **A** and **B**, USA, and Germany (Pearson's correlation).

Clinical significance of the bFDRP‐SLOV was tested by correlating pattern's expressions (*z*‐scores) with MMSE and disease duration (in Cohorts Slovenia **A**, **B**, USA, and Germany).

### 2.6 Longitudinal analysis

To explore changes in bFDRP‐SLOV in time, we investigated a longitudinal cohort Slovenia **D** (Table 1), consisted of patients that underwent FDG‐PET imaging twice (timespan between the two scans 3.0 ± 2.0 years). Changes in bFDRP‐SLOV in time were explored with linear mixed model with disease duration as fixed and patient ID as a random variable.

**TABLE 1:** Demographic characteristics of subjects

		Slovenia					
				Slovenia D		USA	Germany
		Slovenia A	Slovenia B	TP1	TP2	USA	Germany
bvFTD	*N* (M/F)	20 (10/10)	20 (8/12)	6 (3/3)		25 (16/9)	44 (35/9)
	Age	66.5 ± 10.8	71.0 ± 8.9	65.7 ± 6.6	68.4 ± 7.1	70.7 ± 10.5	63.1 ± 9.4
	Disease duration	2.8 ± 2.2	3.4 ± 2.7	2.2 ± 2.2	4.8 ± 3.0	3.0 ± 1.8	3.7 ± 3.1
	MMSE	19.3 ± 3.7	21.0 ± 5.0	27.8 ± 1.9	19.0 ± 1.0	25.2 ± 3.9	22.8 ± 4.8
	CSF availability	15/20	15/20	‐	‐	‐	15/44
	CSF tau	387 ± 123	420 ± 164	‐	‐	‐	361 ± 266
	CSF *p*‐tau	45 ± 9	56 ± 18	‐	‐	‐	‐
	CSF amyloid‐beta	1159 ± 252	1057 ± 317	‐	‐	‐	790 ± 379
HC	*N* (M/F)	20 (8/12)	20 (7/13)			22 (12/10)	15 (7/8)
	Age	66.7 ± 5.7	63.0 ± 9.1			69.7 ± 6.4	61.8 ± 9.1
	MMSE	28.6 ± 1.2	29.1 ± 1.3			‐	‐

		Slovenia C				Germany
AD	*N* (M/F)	26 (14/12)	svPPA	8 (2/6)	svPPA	16 (10/6)
	Age	74.3 ± 9.8		65.1 ± 10.8		65.4 ± 4.4
	Disease duration	3.9 ± 1.9		3.2 ± 1.8		3.8 ± 2.2
	MMSE	19.0 ± 4.9		18.6 ± 5.7		22.3 ± 6.1
	CSF availability	26/26		7/8		‐
	CSF tau	712 ± 253		292 ± 116		‐
	CSF *p*‐tau	94 ± 24		41 ± 15		‐
	CSF amyloid‐beta	568 ± 94		1096 ± 82		‐
sCJD	*N* (M/F)	16 (7/9)	nfvPPA	6 (5/6)	nfvPPA	12 (6/6)
	Age	68.1 ± 10.7		68.6 ± 8.6		70.2 ± 8.9
	Disease duration	0.4 ± 0.4		4.8 ± 3.3		3.5 ± 1.8
	MMSE	15.0 ± 7.8		25.8 ± 1.3		20.9 ± 6.4
	CSF availability	15/16		4/6		‐
	CSF tau	2172 ± 347		222 ± 95		‐
	CSF *p*‐tau	63 ± 23		42 ± 17		‐
	CSF amyloid‐beta	1078 ± 419		1086 ± 281		‐

*Note*: Number of patients (*N*), gender (M, male; F, female), age at FDG‐PET, disease duration, Mini‐Mental State Examination (MMSE) scores and cerebrospinal fluid (CSF) biomarker results of all the studied cohorts are presented with mean and SD.

Abbreviations: AD, Alzheimer's disease; bvFTD, behavioral variant of frontotemporal dementia; HC, healthy controls; nfvPPA, nonfluent variant of primary progressive aphasia; sCJD, sporadic Creutzfeldt‐Jakob's disease; svPPA, semantic variant of primary progressive aphasia; TP1, time point 1; TP2, time point 2.

### 2.7 Internal network organization

To explore bFDRP‐SLOV internal network organization, we applied graph‐theory methods (Ko et al., 2018; Niethammer et al., 2018; Schindlbeck et al., 2020). The bFDRP‐SLOV map was divided into 95 regions of interest (nodes) according to AAL atlas as described previously (Ko et al., 2018). Regional bFDRP‐SLOV weights of the nodes were then *z*‐transformed and the most hyperactive or hypoactive nodes (|*z*| > 1) further entered the connectivity analysis. The voxel‐based bFDRP‐SLOV was therefore transformed into 17 hyperactive and 14 hypoactive nodes.

For each node, metabolic activity was calculated in HC and bvFTD subjects from cohorts Slovenia **A** and **B**, USA, and Germany and normalized to pons as described elsewhere (Ko et al., 2018). Metabolic data from each site were then used to construct node‐to‐node correlation matrices separately for HC and bvFTD. Further, an in‐house script was used to generate 100 bootstrap samples for each group (site × diagnosis) and the median node‐to‐node correlation pairs were used to construct a reliable matrix of each group's graph. Relative influence of each node in the corresponding graph was expressed by a graph theoretic measure eigenvector centrality as a median value of 100 bootstrap iterations for each node. Correlation coefficients were determined by the Statistics and Machine Learning Toolbox in MATLAB R2021b. Graphs were visualized in BrainNet toolbox (Xia et al., 2013) in MATLAB 2021b.

Agreement (concordance) between bvFTD patient correlational matrices from the three sites was explored using two‐way mixed, absolute, single measure intraclass correlation (Cicchetti, 1994; Hallgren, 2012).

Connectivity changes in bvFTD in cohorts Slovenia **A** and **B** were explored by comparing all the connection pairs between HC and bvFTD subjects as described elsewhere (Schindlbeck et al., 2020). A connection was considered gained or lost if the absolute correlation coefficient (connection) in any graph (bvFTD or HC) exceeded |*r*| > .6 (*p* < .05), the difference in such coefficient between the two graphs exceeded |*∆r*| > .4 (*p* < .05, permutation test, 1000 iterations) and the connections that satisfied these criteria were confirmed by bootstrap resampling (100 iterations).

Differences in global connectivity between bvFTD and HC subjects from all three sites were further studied by comparison of several connectivity measures calculated using Brain Connectivity Toolbox (Rubinov & Sporns, 2010): degree centrality, random graph normalized clustering coefficient, random graph normalized characteristic path length, small‐worldness, and assortativity (Schindlbeck et al., 2020; Vo et al., 2022). These measures were calculated at multiple node‐to‐node correlation thresholds (between |*r*| = .30 and |*r*| = .60 with .05 increments) for all the 100 bootstrap iterations and compared between bvFTD and HC using two‐way repeated measure ANOVA for each site independently.

### 2.8 Exploring the relationship between atrophy and metabolic bFDRP‐SLOV pattern

While atrophy is a universal finding in bvFTD, we explored its relationship with bFDRP‐SLOV.

A subset of 12 Slovenia **A** and **B** bvFTD patients (4 from Slovenia **A** and 8 from Slovenia **B**, 5 males and 7 females, age 65.3 ± 9.4 years, disease duration 2.6 ± 1.2 years, MMSE 20.7 ± 5.0) and 12 age‐matched HC (3 males, 9 females, age 65.1 ± 12.6 years) underwent brain MRI performed on the 1.5T Philips Achieva MR scanner at the Neuroradiology Department, Clinical Institute of Radiology, UMC Ljubljana as a part of routine clinical workup. In these patients, MRI was performed on average 1.9 ± 5.0 months before FDG‐PET. Isomorph 3‐dimensional T1‐weighted images were acquired with either SPGR (echo time = 4.6 ms, repetition time = 25 ms, flip angle = 30°, matrix 240 × 240 × 175; 6 bvFTD and 4 HC scans) or MPGR sequence (echo time = 3.2 ms, repetition time = 7.1 ms, flip angle = 8°, matrix 156 × 256 × 160; 6 bvFTD and 8 HC scans), giving a resolution of 1 mm in all planes.

MRI scans were converted to Analyze format. After spatial normalization using MNI‐based template, gray matter was segmented and smoothed using a three‐dimensional Gaussian kernel of 10 × 10 × 10 mm in SPM5 software (Wellcome Trust Centre for Neuroimaging), running in Matlab 7.0 (MathWorks Inc.).

Similar to the procedure of multivariate pattern identification in metabolic FDG‐PET scans described above, we identified structural multivariate pattern related to atrophy in bvFTD. The SSM/PCA procedure was applied to the 12 bvFTD and 12 HC preprocessed MRI scans. As above, a set of first 6 PCs was further analyzed and entered into series of logistical regression models among which the one with lowest AIC was chosen and used to generate the multivariate bvFTD atrophy related pattern (MRI‐bFDRP). Stability of the pattern was again explored by the bootstrapping procedure described above.

The relationship between metabolic bFDRP‐SLOV and structural MRI‐bFDRP was explored at the topographical level with the voxel‐wise Pearson's correlation between the two patterns (using autocorrelation correction (Ko et al., 2014)) and at the subject level correlating both patterns' *z*‐scored expression values of the 12 bvFTD patients that underwent imaging with both modalities. As above, expression values of both metabolic and structural patterns *z*‐scored against normal controls were compared to assess which pattern predominates.

### 2.9 Relationship between bFDRP‐SLOV and DMN

We first identified the overlapping regions of both bFDRP‐SLOV and previously FDG‐PET‐derived DMN pattern (Spetsieris et al., 2015) (additively inversed as we were exploring DMN loss) with voxel values >1 or <–1 (bFDRP ∩ DMN; Figure 4a) and then the regions specific for bFDRP‐SLOV by excluding DMN voxels with weights >1 or <−1 from the bFDRP‐SLOV (bFDRP\DMN). The relationship between both topographies was explored topographically by voxel‐wise correlation and on a subject scale by correlating both patterns' subject scores from all three sites. Furthermore, to explore whether any pattern predominates, we compared subject scores of the bFDRP‐SLOV pattern and subject scores of DMN loss (*z*‐scored to the same HC) by paired *t*‐test.

To further distinguish the specific bFDRP‐SLOV pattern from the nonspecific DMN loss, we explored the internal structure of DMN loss in HC and bvFTD patients with graph theory. As described above, the FDG‐PET based DMN topographical map (Spetsieris et al., 2015) was transformed into 95 regions of interest and those with regional *z*‐scored weight >1 or <–1 defined the metabolic DMN space, which was comprised of 18 underactive and 21 overactive nodes (Schindlbeck et al., 2020). Regional metabolic activity in these nodes was used to generate separate correlation matrices (100 bootstrap samples) from bvFTD and HC subjects scanned at each of the three sites. Median graphs from each population were visualized and compared in the bFDRP‐SLOV vector space. Graph metrics (degree centrality, random graph normalized clustering coefficient, random graph‐normalized characteristic path length, small‐worldness, and assortativity) were compared for bvFTD and HC subjects scanned at the different sites.

Consistency in bvFTD correlational matrices in DMN space from all three sites was explored using two‐way mixed, absolute, single measure intraclass correlation (Cicchetti, 1994; Hallgren, 2012). To further study the consistency between the three sites in more detail, the DMN and bFDRP‐SLOV spaces were broken down into subnetworks (Figure 4a): common bFDRP‐SLOV and DMN nodes (bFDRP ∩ DMN), the nonspecific DMN nodes that were not part of the bFDRP space (DMN\bFDRP) and exclusive bDRP‐SLOV nodes (bFDRP\DMN; Figure 4a). Consistency of bvFTD correlational matrices was therefore studied (1) in bFDRP ∩ DMN correlation pairs, (2) in DMN\bFDRP correlation pairs, (3) in bFDRP\DMN correlation pairs, and (4) correlation pairs between bFDRP ∩ DMN and DMN\bFDRP nodes to explore whether there is an intercenter variability in bvFTD specific and nonspecific DMN and bFDRP‐SLOV subnetworks.

### 2.10 Statistical analysis and software

Differences in demographic data and pattern expressions among groups were assessed by one‐way ANOVA and post‐hoc Bonferroni's correction. Logistic regression and mixed model analyses were performed on JMP 14 software (SAS Institute Inc., Cary, NC, USA). All other statistical tests were performed in GraphPad Prism 8 for Windows (GraphPad Software, San Diego, CA, USA). For all comparisons, *p* value below .05 was considered statistically significant. SPM12 (Institute of Neurology, UCL, London, UK), BrainNet Viewer (National Key Laboratory of Cognitive Neuroscience and Learning, Beijing Normal University, Beijing, China) and MRIcro software (McCausland Center for Brain Imaging, University of South Carolina, Columbia, SC, USA) were used for visualization of the results.

## 3 RESULTS

### 3.1 Subjects

Demographic data of all the included cohorts are presented in Table 1. bvFTD and HC subjects from all the cohorts (Slovenia **A**, **B**, **D**, USA and Germany) differed in age (*F*_8,183_ = 3.4, *p* = .001, one‐way ANOVA) with Germany bvFTD patients being younger than USA and Slovenia **B** patients (*p* < .04, post‐hoc Bonferroni's test). Subjects also differed in MMSE (*F*_5,143_ = 22.1, *p* < .0001) with lowest scores in both Slovenian bvFTD groups (without difference between them, *p* = .94, post‐hoc Bonferroni's test), followed by Germany bvFTD group (Slovenia A vs. Germany bvFTD: *p* = .01), USA bvFTD group (Slovenia A vs. USA bvFTD: *p* = .0004) and highest scores in HC (Slovenia A bvFTD vs. both HC: *p* < .0001) (Figure S1). We did not find any differences in disease duration among bvFTD groups (*F*_4,110_ = 0.9, *p* = .50).

Considering bvFTD and both PPA subgroup patients from cohorts Slovenia **A**, **B**, and **C** and Germany, there were no difference in age nor in disease duration (both *F*_6,119_ ≤ 1.4, *p* ≥ .22). These groups differed in MMSE scores (*F*_6,119_ = 2.5, *p* = .03) but the post‐hoc analysis did not find significant differences between individual group pairs after multiple comparison correction (all *p* > .13).

### 3.2 bFDRP‐SLOV identification, validation, and clinical correlations

**FIGURE 1:**
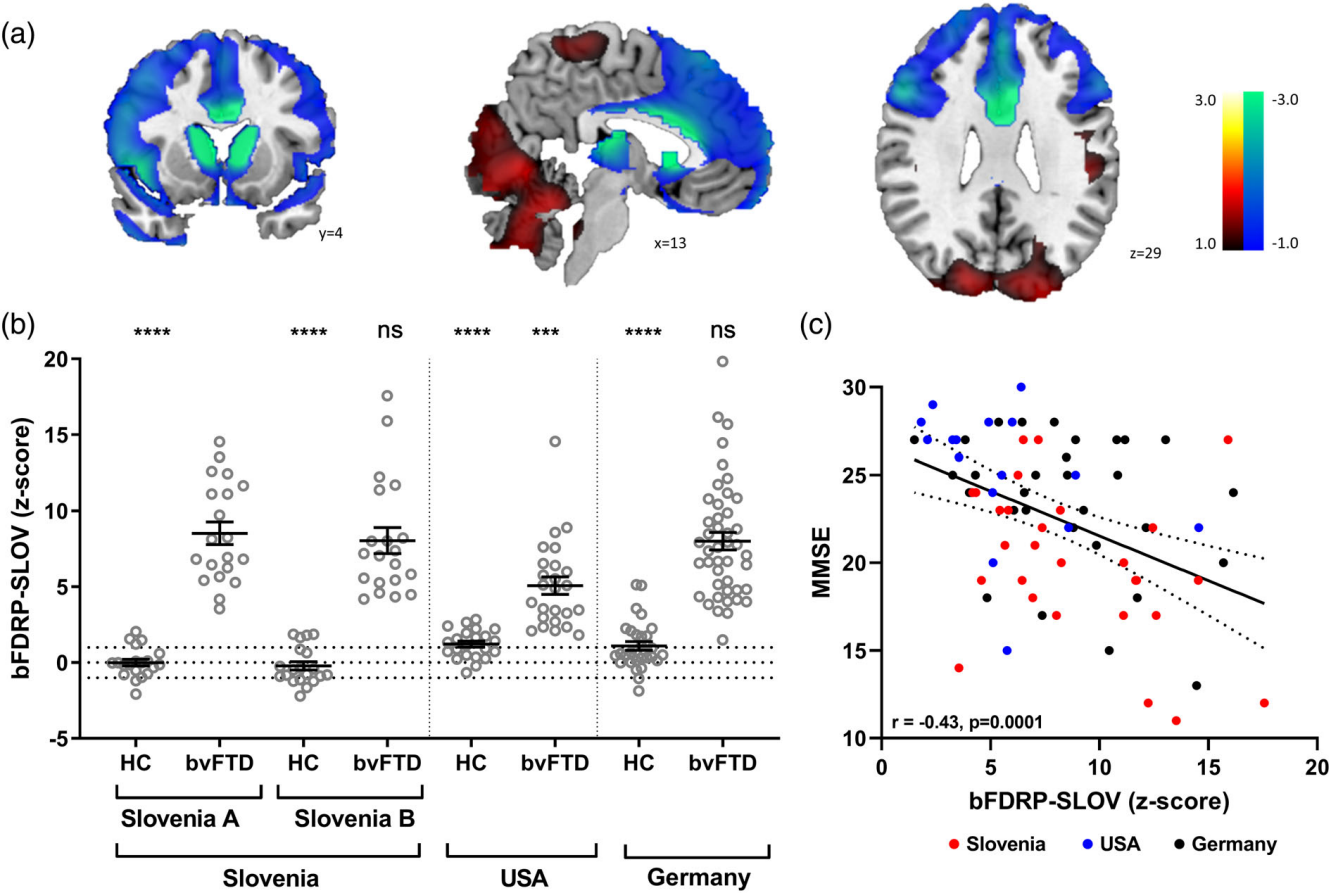
(a) Topography of behavioral variant frontotemporal dementia (bvFTD) related pattern identified in cohort Slovenia a (bFDRP‐SLOV), (b) bFDRP‐SLOV expression scores of bvFTD patients and normal controls (NC) from the identification cohort Slovenia a and validation cohorts Slovenia B, USA, and Germany, and (c) correlation between bFDRP‐SLOV and mini‐mental state examination (MMSE) scores in bvFTD patients from all three sites. (a) bFDRP was identified using multivariate SSM‐PCA analysis of FDG‐PET images from 20 probable bvFTD patients and 20 age‐matched HC. Relative metabolic decrease is color‐coded blue and relative metabolic increase red. Coordinates in the axial (*z*), coronal (*y*), and sagittal (*x*) planes are in Montreal Neurological Institute (MNI) standard space. (b) Mean values and standard errors are presented. Vertical lines separate cohorts from different centers. One‐way ANOVA among all groups (including other dementia disorders—see text and Figure S3) was performed (*F*_14,271_ = 27.0, *p* < .0001) and results of post‐hoc Bonferroni's multiple comparison test between identification bvFTD group and other groups are presented (***p < .001, ****p < .0001, ns: *p* > .05). (c) Linear regression line with 95% confidence interval is presented.

The first six PCs from the SSM/PCA analysis in the identification Slovenia **A** cohort represented 65.3% of the total subject × region variance. In a series of logistic regression models, the model with PC1 (33.2% of total variance) achieved the lowest AIC and optimally distinguished bvFTD from HC (*χ* ^2^ = 55.5, *p* < .0001). Therefore, the PC1 was identified as the bFDRP‐SLOV metabolic pattern, which was characterized by relative hypometabolism in the anterior and middle cingulate, inferior, middle and superior frontal gyri, orbital gyri, insula, caudate and thalamus bilaterally and in temporal pole and superior temporal gyrus on the left side. Relative hypermetabolism was observed in limited occipital and cerebellar regions (Figure 1a, Table S1). All the regions were proven stable by the bootstrapping procedure at *p* < .05 (Figure S2).

To prospectively validate the pattern, bFDRP‐SLOV expression values (*z*‐scored) of bvFTD and HC subjects from cohorts Slovenia **A**, **B**, USA, and Germany were compared (Figure 1b). bFDRP‐SLOV subject scores highly exceeded those of corresponding HC in each of the four cohorts (*p* < .0001, Bonferroni's correction for multiple comparisons). bFDRP‐SLOV scores were similarly high (*p* > .9999) among bvFTD patients from the Slovenian **A**, **B**, and Germany cohorts, although bvFTD patients in the identification Slovenia **A** cohort had higher values (*p* = .0003) than those from the USA cohort. However, the difference in bFDRP expression scores among bvFTD groups became nonsignificant after the MMSE effect was controlled in the analysis (one‐way ANOVA: *F*_3,89_ = 1.0, *p* = .40; post‐hoc comparison between all groups, *p* ≥ .51, Bonferroni's correction).

Furthermore, bFDRP‐SLOV subject scores were also calculated in AD, CJD, svPPA, and nfvPPA patients from Slovenia **C** and Germany cohorts (Figure S3). Pattern scores in the Slovenia **A** bvFTD patients were significantly higher than those from the AD, sCJD, svPPA, and nfvPPA patients in Slovenia **C** (all comparisons; *p* < .0001, post‐hoc Bonferroni's). Likewise, in the German cohort, the pattern's expression of the bvFTD group exceeded that of the svPPA and nfvPPA (*p* < .0004, post‐hoc Bonferroni's test).

The topography of the newly identified bFDRP‐SLOV was similar to the previously reported bFDRP‐NY by both visual comparison (Figure S4A) and the voxelwise correlational analysis showing high topographical correlation between the two patterns (*r* = .86, *p* < .001, corrected for spatial autocorrelation, Figure S4B). Similarly, we observed a high correlation of subject scores for the two bFDRPs was even higher (*r* = .96, *p* < .0001, Figure S4C).

The bFDRP‐SLOV expression correlated inversely with MMSE scores in the combined group of Slovenia **A**, **B**, USA, and Germany bvFTD patients (*n* = 109; *r* = –.42, *p* = .0001) (Figure 1c). A weak trend of positive correlation, albeit nonsignificant (*r* = .18, *p* = .07), was found between pattern expression and disease duration in these patients.

### 3.3 Longitudinal analysis

**FIGURE 2:**
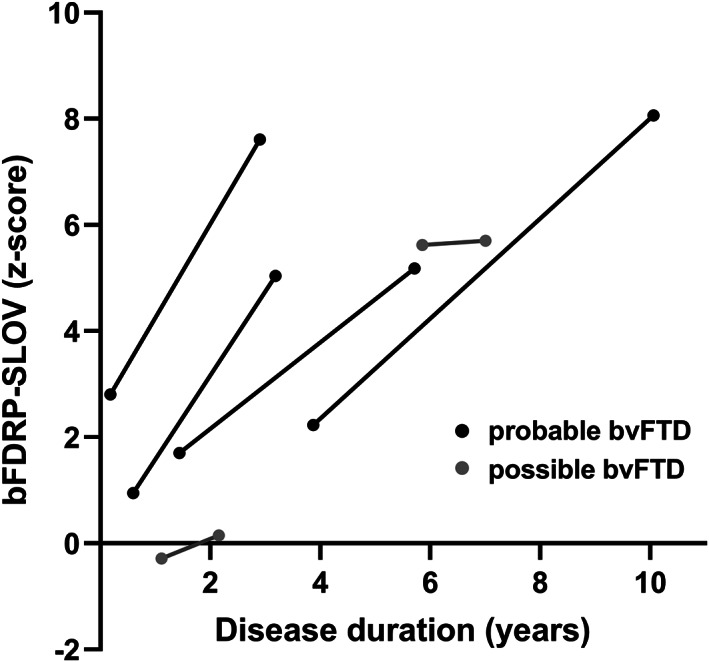
Longitudinal analysis of bvFTD patients that underwent FDG‐PET twice. Lines represent changes in bFDRP‐SLOV expression scores between the two scans in individual patients. Clinical diagnosis of probable or possible bvFTD was based on Rascovsky et al. (2011).

In the longitudinal cohort Slovenia **D**, bFDRP‐SLOV expression scores rose between the two scans of all the six bvFTD patients (*p* = .02, paired *t*‐test). Mixed regression model further affirmed significant increase in bFDRP‐SLOV over time (annual *z*‐scored increase in bFDRP‐SLOV = 0.95; 95% CI 0.57–1.33, *p* = .0007) (Figure 2).

### 3.4 bFDRP‐SLOV internal structure

**FIGURE 3:**
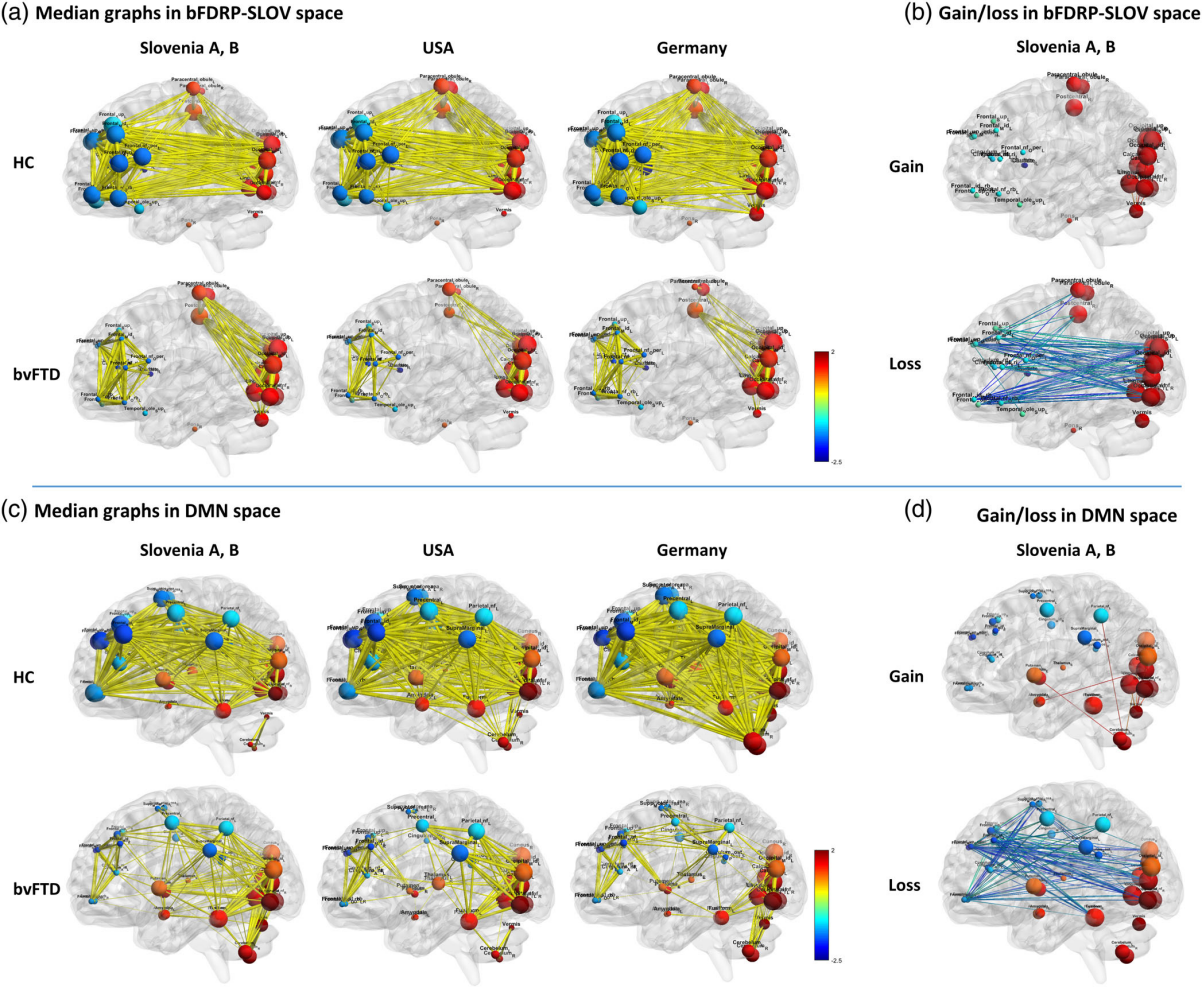
Functional connectivity within (a) bFDRP‐SLOV and (c) default mode network (DMN) vector space in all the three populations from Slovenia, USA, and Germany. The right panels (b and d) represent gain and loss of connections in bvFTD patient compared to HC in patients from Slovenia. Functional connectivity is presented for normal controls (NC) and behavioral variant frontotemporal dementia (bvFTD) patients. Reorganization of network is seen in both network spaces; however, in bFDRP‐SLOV space (a) the network is completely disconnected while in the DMN space (c) frontal areas remain connected with other parts of the network through middle/posterior cingulate, precentral, and supramarginal nodes. Edge thickness corresponds to the magnitude of the correlation. All the edges exceeding |*r*| > .6 are presented. (b, d) Analysis of gain and loss of connection between HC and bvFTD in bFDRP‐SLOV and DMN vector spaces presented on data from cohorts Slovenia A and B. (Nodes are color‐coded according to the CJDRP weights; the diameter of nodes corresponds to eigenvector centrality [see Section 2].)

**FIGURE 4:**
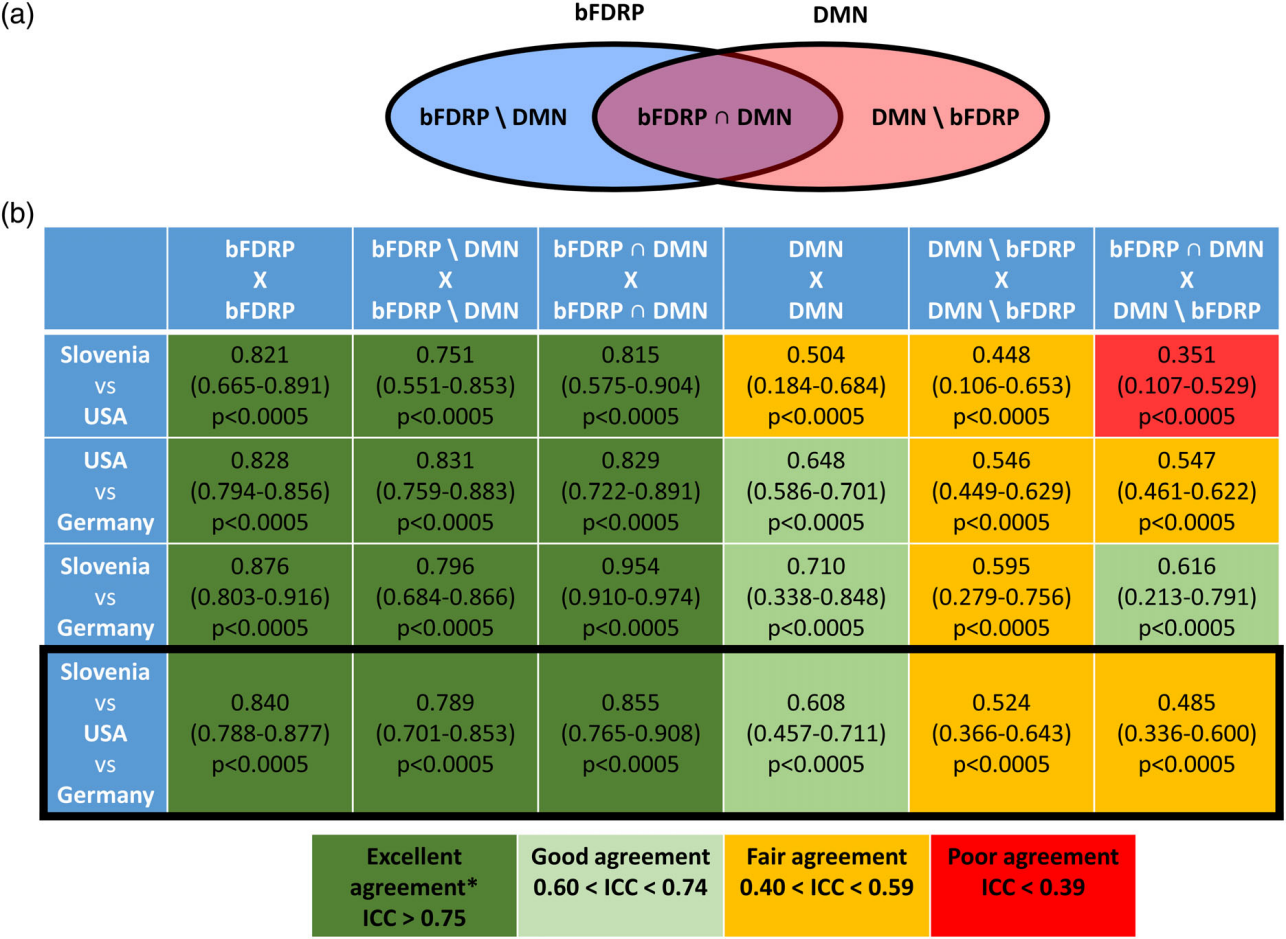
(a) Scheme representing topographic overlay between bFDRP and DMN defining subnetwork spaces and (b) consistency of correlational matrices in different network subspaces across the sites. (A) DMN—default mode network, bFDRP\DMN—regions specific for bFDRP and not included in DMN, bFDRP ∩ DMN—regions overlapping bFDRP and DMN, DMN\bFDRP—regions specific for DMN and not included in bFDRP. (b) Consistency in correlational matrices across the three sites was explored using two‐way mixed, absolute, single measure intraclass correlation (Cicchetti, 1994; Hallgren, 2012).

The internal structure (connectivity) within the bFDRP‐SLOV defined space was studied in bvFTD and HC subjects from all the three sites (Slovenia A and B subjects were combined) (Figure 3a). Median graphs derived from 100 bootstrap correlation pairs showed prominent disruption of the network in bvFTD patients, which was split into two unconnected modules at the cutoff |*r*| = .60: fronto‐temporal and parieto‐occipital. The concordance in connectivity matrices among sites was excellent (ICC = 0.84, *p* < .0005) (Figure 4). Further analysis confirmed pronounced loss of connections between fronto‐temporal and parieto‐occipital modules and sparse gain of connections within occipito‐vermal part of parieto‐occipital module (Figure 3b). Furthermore, extensive decrease in eigenvector centrality was noted in the frontotemporal module in contrast to parieto‐occipital module (Figure 3a).

Comparing bvFTD connectivity measures with respect to HC on a whole network scale, a highly significant decrease in degree centrality and increase in random graph normalized clustering coefficient, characteristic path length, small‐worldness and assortativity was found in all the cohorts studied (*p* < .0001) (Figure S5).

### 3.5 Relationship between atrophy and metabolic bFDRP‐SLOV pattern

Atrophy‐related structural pattern MRI‐bFDRP was identified in a subset of Slovenia **A** and **B** bvFTD patients and a new cohort of age‐matched HC. This subgroup did not differ from the rest of bvFTD Slovenia **A** and **B** patients in age, disease duration nor in bFDRP‐SLOV expression (*p* > .11, Student's *t*‐test).

The SSM‐PCA procedure resulted in a set of PCs. After reviewing PCs topographies, PC1 was excluded as it consisted of preprocessing‐related artifacts. The following six PCs entered a series of logistic regressions and a linear combination of PC3 and PC5 (accounting for 8.7% and 6.0% subject × voxel variance) was found to optimally distinguish between bvFTD and HC (*χ* ^2^ = 11.2, *p* = .004).

The MRI‐bFDRP (Figure S6A) was characterized by atrophy in bilateral anterior cingulate, inferior, middle, and superior frontal gyri, supplementary motor area, insula, hippocampi, caudate and putamina, left temporal pole, and left inferior parietal gyrus (significant regions on the *z*‐scored map, voxel size >70, *p* < .05, one sided). All these regions were stable on bootstrapping procedure at *p* < .05 (Figure S6B).

The structural atrophy‐related MRI‐bFDRP topographically correlated with the metabolic bFDRP‐SLOV pattern (*r* = .55, *p* < .001, corrected for autocorrelation) (Figure S6C). There was also a trend of correlation on a subject score level but did not reach a significant level (*r* = .54, *p* = .07). *z*‐scored values were considerably more expressed in the metabolic bFDRP‐SLOV compared to the structural MRI‐bFDRP pattern (*p* < .0001, paired *t*‐test, Figure S6D).

### 3.6 Relationship between disease specific bFDRP‐SLOV and nonspecific DMN

**FIGURE 5:**
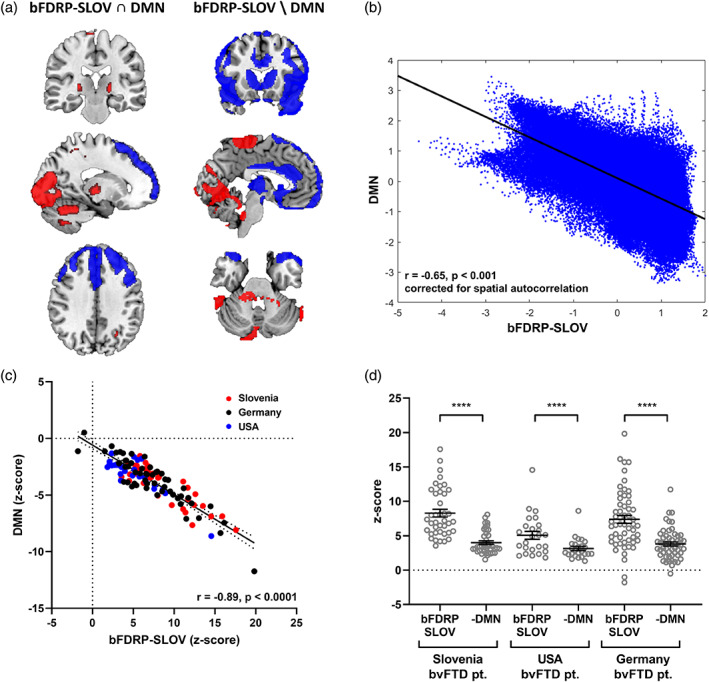
Comparison between bFDRP‐SLOV and default mode network (DMN). (a) left: Topography of overlapping areas of bFDRP‐SLOV and DMN patterns (overlapping hypoactive bFDRP‐SLOV and DMN loss areas are color‐coded blue, overlapping hyperactive bFDRP‐SLOV areas and DMN‐non‐affected areas are color‐coded red); right: topography of bFDRP‐SLOV specific regions after DMN regions were excluded. (b) Significant correlation between bFDRP‐SLOV and DMN topographies (*r* = −.65, *p* < .001, corrected for spatial autocorrelation) and (c) subject scores derived from both patterns (*r* = −.89, *p* < .0001). (d) bvFTD patients' expression scores calculated on both patterns (bFDRP‐SLOV and DMN loss) and standardized (*z*‐scored) to same healthy controls significantly differed in all three sites (****p < .0001, paired *t‐*test).

bFDRP‐SLOV overlapped with DMN in anterior and middle cingulum, superior, middle and inferior gyri, and orbital gyri (relative hypoactivity/DMN loss). Relative preservation of metabolism was common to both patterns in the occipital cortex, cerebellum, and putamina (Figure 5a, left). Hypometabolic areas specific for bFDRP not included in the DMN pattern were found in caudate, thalami, temporal poles, and insular cortex bilaterally, while areas with preserved metabolism in bFDRP‐SLOV in contrast to DMN loss were located in paracentral lobule, pre‐ and postcentral gyri, superior parietal gyri, and precunei (Figure 5a, right). bFDRP‐SLOV significantly correlated with DMN loss topographically (*r* = –.65, *p* < .001, corrected for autocorrelation, Figure 5b) and on a subject score level (*r* = .89, *p* < .0001, Figure 5c). However, the expression of disease specific bFDRP‐SLOV pattern was significantly higher compared to the nonspecific DMN loss in all three sites (both patterns' subject scores were *z*‐scored to the same HC) (*p* < .0001, paired *t*‐test, Figure 5d).

Metabolic connectivity analysis in bvFTD and HC subjects within the DNM defined vector space revealed different networks than the one in the bFDRP‐SLOV space. In contrast to bFDRP‐SLOV space, where the network was completely disconnected into two modules (Figure 3a), the DMN defined network remained connected through middle/posterior cingulate, precentral and supramarginal nodes (Figure 3c). In the anterior hypoactive DMN nodes (frontal, orbitofrontal, anterior cingulum) a marked decrease in eigenvector centrality in bvFTD patients was observed, similar to that in the hypometabolic regions of the bFDRP‐SLOV network. On the contrary, in the posterior hypoactive DMN nodes (precentral, parietal, supramarginal) this change in eigenvector centrality was not so pronounced. Similar to the bFDRP‐SLOV network, the loss of connections was found predominantly between frontal and occipito‐parietal nodes and a sparse gain predominantly within occipito‐cerebellar regions.

On a whole network scale, differences in DMN space connectivity measures between bvFTD and HC were similar to those seen in the bFDRP‐SLOV network: a highly significant decrease in degree centrality and increase in clustering coefficient, characteristic path length, small‐worldness, and assortativity were found in all the cohorts studied (*p* < .0001) (Figure S7).

Consistency in correlational matrices in DMN space between the three sites was good (ICC = 0.61, *p* < .0005); however, it was considerably lower compared to those in the bFDRP‐SLOV space (Figure 4b). By dividing DMN space into a disease specific subnetwork with nodes common to both DMN and bFDRP‐SLOV (bFDRP ∩ DMN, Figure 4a) and nonspecific subnetwork that consisted of exclusive DMN nodes (DMN \ bFDRP), we showed excellent agreement in the disease specific bFDRP ∩ DMN space but only fair agreement in other nonspecific connection pair matrices (Figure 4b). On the other hand, correlational consistency at all sites was excellent in the portion of bFDRP‐SLOV that was outside the DMN space.

## 4 DISCUSSION

In the present study, we identified the multivariate metabolic brain pattern specific for bvFTD (bFDRP‐SLOV) in a new cohort of probable bvFTD patients. We prospectively validated it in three independent bvFTD cohorts and other dementia patients, as well as demonstrated its correlation with cognitive decline. We have also shown concordance of this pattern with the one published previously (Nazem et al., 2018). Further longitudinal analysis in a small subset of bvFTD patients suggested that the pattern might be used as a marker of disease progression. The connectivity studies have revealed complete disruption of the pattern's internal structure with disconnection between frontotemporal and parieto‐occipital modules. We have further shown that while atrophy is universally present in bvFTD, its effect is relatively low compared to extensive frontotemporal hypometabolism. Finally, we showed that gain of bFDRP‐SLOV coexists with DMN loss in bvFTD patients. While both patterns topographically overlap, we were able to demonstrate that the bFDRP is indeed independent and dominates over DMN loss in bvFTD patients.

Despite that the topography of metabolic brain changes in bvFTD has been well characterized in the past in several univariate studies (Cerami et al., 2016; Diehl‐Schmid et al., 2007; Foster et al., 2007; Rascovsky et al., 2011) and one multivariate study (Nazem et al., 2018), the current bFDRP‐SLOV substantiated these findings extensively in a large, multicenter group of 111 bvFTD patients. Topography of bFDRP‐SLOV was indeed found to be very similar to the previously identified multivariate pattern (Figure S4A). In contrast to univariate approaches, the major advantage of the SSM/PCA is its ability to prospectively measure pattern expression in individual subjects. While there were no differences in bFDRP‐SLOV expression among the Slovenian and Germany bvFTD groups, the relatively lower bFDRP‐SLOV subject scores in USA bvFTD patients may be due to their clinical characteristics of being less cognitively impaired than patients in other groups (Figure S1). Comparison of bvFTD patients with other dementia syndromes (i.e., AD, sCJD, svPPA, and nfvPPA) showed that the mean *z*‐scored bFDRP‐SLOV values in the bvFTD groups were around 5.0–8.0, while all other dementia groups had lower expression levels between 2 and 3.9. Nonetheless, the bFDRP‐SLOV expression values of other dementia syndromes evidently still exceeded those of the HC. These phenomena may be due to partial overlap of abnormal networks involved in different neurodegenerative disorders which have been observed in many prior studies (Ge et al., 2018; Ko et al., 2016; Rus et al., 2020; Shen et al., 2020). Indeed, for reliable single case diagnosis several metabolic network expression must be considered (Rus et al., 2020; Tang et al., 2010).

In contrast to the previous smaller multivariate study, we were able to show a highly significant correlation between bFDRP‐SLOV expressions and cognitive decline measured by MMSE. However, the MMSE was able to explain only 18.7% of total bFDRP‐SLOV variance, which is not unexpected given that MMSE was developed as a screening cognitive test and has been rigorously tested in AD as it focuses on cognitive domains predominantly impaired in AD (working memory, memory recall, orientation, and visuospatial functions) (Arevalo‐Rodriguez et al., 2015). Although the domains specific for bvFTD such as executive functions, behavior, and language are less emphasized in MMSE, it was shown that MMSE is still a reliable but nonspecific screening tool for cognitive decline in bvFTD patients (Chow et al., 2006). However, as MMSE does not address the most affected cognitive domains in bvFTD, it may explain only a minor part of overall bFDRP‐SLOV variance. Unfortunately, detailed neuropsychological status of our cohorts was not available. While pattern's expression correlated with cognitive decline, only a trend of correlation was found with disease duration. This may be partially due to diversity of disease duration associated with genotypes/phenotypes (Moore et al., 2020) and partially due to less reliable and subjective history of disease onset.

In addition to replicating the pattern and showing its clinical correlates, we were able to demonstrate its role as a disease progression marker. Although the longitudinal analysis was performed in a very small sample, the results showed significant progression in bFDRP‐SLOV expression over time both on the group‐mean level (i.e., approximately one *z*‐scored point increase per year) and individually in all six studied patients.

Until recently, the multivariate metabolic brain patterns could only be studied as discrete networks of brain areas characterized by metabolic changes without any knowledge of their internal connectivity structure. However, recent advances in graph theory methods have given us in‐depth insight into individual metabolic network vector space. Using these techniques, we were able to reveal a pronounced network disruption in the bFDRP‐SLOV space consistent across all the three sites with excellent intercenter agreement. The global connectivity measures showed lower degree centrality in bvFTD compared to HC corresponding with overall loss of connections, which was mostly expressed in frontotemporal nodes (compare node diameters representing eigenvector centrality in Figure 3a) and high clustering coefficient indicating higher local density. The higher path length (average length of shortest paths between all possible node pairs) and especially assortativity (tendency for network connections to link nodes with similar degree centrality) indicated general inefficiency of information transfer through the network. These connectivity measures agree with previous resting state fMRI results, which explored whole brain rather than network specific subspaces (Agosta et al., 2013). The previous fMRI study, in line with our metabolic data, showed pronounced loss of connections between frontal regions on one side and occipital and temporal regions on the other. On a global scale (in the whole brain space), degree centrality was lower and characteristic path and assortativity of the network elevated. These results seem to be conserved across modalities; increase in network assortativity in bvFTD was noticed also in resting state EEG (de Haan et al., 2009). A recent whole brain metabolic connectome study by sparse inverse covariance estimation (SICE) analysis in bvFTD patients similarly showed pathological reconfiguration of connectome especially in the frontal, insular and temporal regions. However, local connectivity changes somehow differed from ours, which may be due to different study design and vector space of exploration (Malpetti et al., 2019).

Despite being causally related and topographically corresponding to metabolic changes in neurodegenerative disorder (Jack et al., 2018; Rascovsky et al., 2011), the atrophy may potentially affect the disease specific patterns. Namely, atrophic areas with enlarged CSF spaces may be erroneously recognized as hypometabolic gray matter by software algorithms. To avoid this potential issue, we identified an atrophy‐related MRI‐bFDRP pattern and explored its relationships with the bFDRP‐SLOV. As expected, the atrophy pattern corresponded well to the metabolic network, despite that expression of the atrophy pattern was significantly less expressed in the same patients on a standardized *z*‐scale. These findings are consistent with previous univariate results proving that metabolic changes are more pronounced and precede atrophy (Garrett & Niccoli, 2022), a finding that was well documented also in other neurodegenerative disorders (Jack et al., 2013). Cortical atrophy needs to be considered in the interpretation of metabolic patterns associated with neurodegenerative disorders such as bvFTD. That said, rigorous 3‐dimensional MRI‐PET registration methods (Lu et al., 2021; Samuraki et al., 2007) is needed to measure the precise contribution of regional volume loss to the PET‐based bFDRP topography. Regrettably, an analysis of this sort was not possible in the current study but is being planned for the future.

Finally, we studied the relationship between disease specific metabolic pattern and nonspecific metabolic DMN loss in bFTDP. Both patterns were found to be affected in bvFTD and, moreover, correlated topographically and on a subject‐score level. These findings are novel, but also raise the question whether the bFDRP‐SLOV is indeed a disease specific network or just a representation of DMN loss. However, further analysis showed that bFDRP‐SLOV includes several specific topographic regions in addition to those overlapped with the DMN (Figure 5). Using graph theory methods to evaluate the relationship between the two networks, we found that the general patterns of gain and loss (Figure 3b,d) and global connectivity measures (Figures S5 and S7) were somewhat similar in both vector spaces. Nonetheless, while the DMN network stayed connected, the bFDRP‐SLOV vector space was completely disrupted in bvFTD patients. Further, in the DMN vector space, the eigenvector centrality was reduced almost exclusively in hypoactive nodes overlapping with bFDRP‐SLOV space (Figure 3c). This overlapping region (bFDRP ∩ DMN) also showed excellent intercenter reliability compared to nonoverlapping DMN regions (Figure 4). That being said, the metabolic connectivity changes in bFDRP‐SLOV space seem to be very robust and repeatable across populations in contrast to the nonoverlapping DMN (DMN\bFDRP; see Figure 4). Overlapping‐anterior DMN thus seems to be incorporated into bFDRP. Disruption of anterior DMN in bvFTD has also been found in previous studies. In metabolic FDG‐PET scans, Malpetti and colleagues recently described extensive connectivity alterations of several fMRI defined core RSN networks using seed‐based interregional correlation analysis (IRCA) (Malpetti et al., 2019), anterior DMN being among the most affected ones. However, due to methodological differences these results are not directly comparable with ours. As our approach employed metabolic data explored by SSM/PCA‐based approach, we did not apply other approaches such as IRCA to explore other core fMRI defined RSN.

Despite that this study explored functional metabolic brain changes from different angles in multiple populations, our results are a subject to some limitations. Patients were recruited from routine clinical settings and regular therapy was not discontinued before imaging. Few of them were receiving small doses of neuroleptic medications that may have limited effect on brain metabolism. However, neuroleptics can cause metabolic increases in the basal ganglia and thalamus (Berti et al., 2014). Such findings, however, were not present in our study, suggesting that the bFDRP was not driven by these effects. The longitudinal cohort is small which limits its generalizability despite significant results. Longitudinal research with larger samples is thus needed. Further, the atrophy‐related MRI‐bFDRP was also identified in a relatively small subgroup of bvFTD patients undergoing diagnostic high resolution MRI with two different sequences (although similar and balanced across patients and HC). While the atrophy pattern obtained in this way may be used for direct comparison with the metabolic pattern, extensive validation would be needed before it could be used in a prospective manner. Finally, to understand the relative contribution of individual core RSN in the bFDRP network and generalize the pattern across modalities, further resting state fMRI studies employing independent component analysis are warranted.

## 5 CONCLUSIONS

In summary, the findings of this study have validated the bFDRP network in a large, independent population and further revealed its internal structure. The bFDRP was demonstrated to be unaffected by structural atrophy and, more importantly, independent of loss of normal RSN. In contrast to the nonspecific DMN loss, connectivity changes in specific bFDRP space were proven to be highly consistent irrespective of population, scanner, and other factors.

## FUNDING INFORMATION

The aspects of this work were supported by the Slovenian Research Agency through the research programme P1‐0389, research project J7‐2600. T.R. is a recipient of the Fulbright Foreign Student Program sponsored by the U.S. Department of State's Bureau of Educational and Cultural Affairs.

## CONFLICT OF INTEREST

The authors report no competing interests with regard to the submitted manuscript. Outside the submitted manuscript DE serves on the scientific advisory boards of and has received fees from The Michael J. Fox Foundation for Parkinson's Research and Ovid Therapeutics, receives consulting fees from MeiraGTx, has received grants from NIH (NINDS, NIAID), and is the coinventor of patents re: Markers for use in screening patients for nervous system dysfunction and a method and apparatus for using same, without financial gain. TG received consulting fees from Abbvie, Alector, Anavex, Biogen, Eli Lilly, Functional Neuromodulation, Grifols, Iqvia/Quintiles, Novo Nordisk, Noselab, NuiCare, Roche Diagnostics, Roche Pharma, Toyama, UCB, and Vivoryon; lecture fees from Biogen, Life Molecular Imaging, Novo Nordisk, Roche Pharma, and Schwabe; and grants to his institution from Actelion and Novartis.

## Supporting information


**Appendix S1** Supporting Information

## Data Availability

The data that support the findings of this study are available from the corresponding author, TR, upon reasonable request.
