# Supplementary material for: Disease specific and nonspecific metabolic brain networks in behavioral variant of frontotemporal dementia
Source: Hum Brain Mapp. 2022 Nov 5;44(3):1079–93. doi: 10.1002/hbm.26140 (PMC9875921; doi:10.1002/hbm.26140)
Supplement: Supplementary file 1 — Appendix S1 Supporting Information [file HBM-44-1079-s001.docx]

**Supplementary Figures**


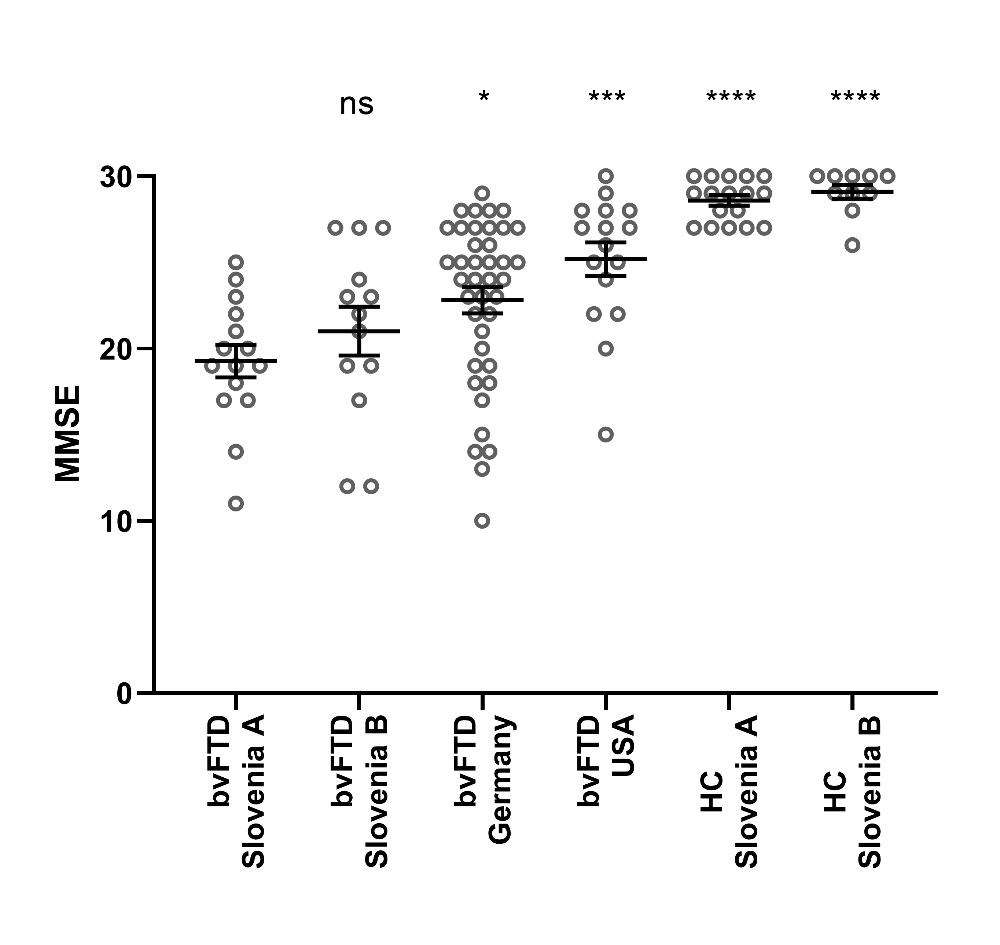


**Figure S1. Distribution of Mini-Mental State Examination (MMSE) scores across bvFTD and NC groups.**

Behavioral variant of frontotemporal dementia (bvFTD groups) and normal control subjects (NC) differed in MMSE scores (one-way ANOVA F_5,105_ = 14.5, p<0.0001). Post-hoc Bonferroni’s multiple comparison test between identification bvFTD group and other groups are presented. [* - p < 0.05, *** - p < 0.001, **** - p < 0.0001, ns – p > 0.05.]


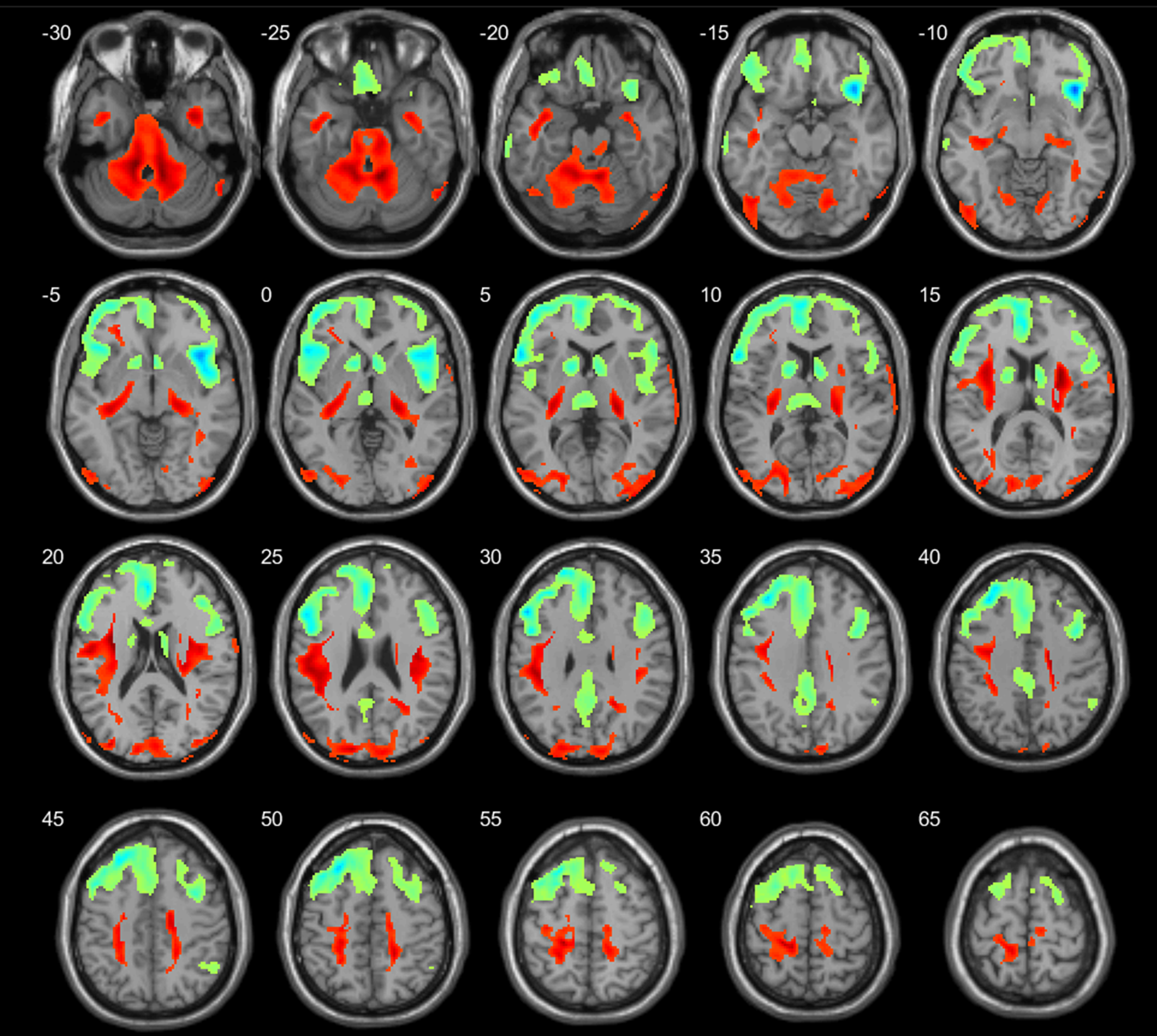


**Figure S2. Results of the bFDRP-SLOV bootstrapping test.**

Stable metabolic brain areas defined by bootstrapping test are presented, 1000 iterations, p<0.05 (z=1.64, one-tail). Relative metabolic decrease is color-coded blue to green and relative metabolic increase red.


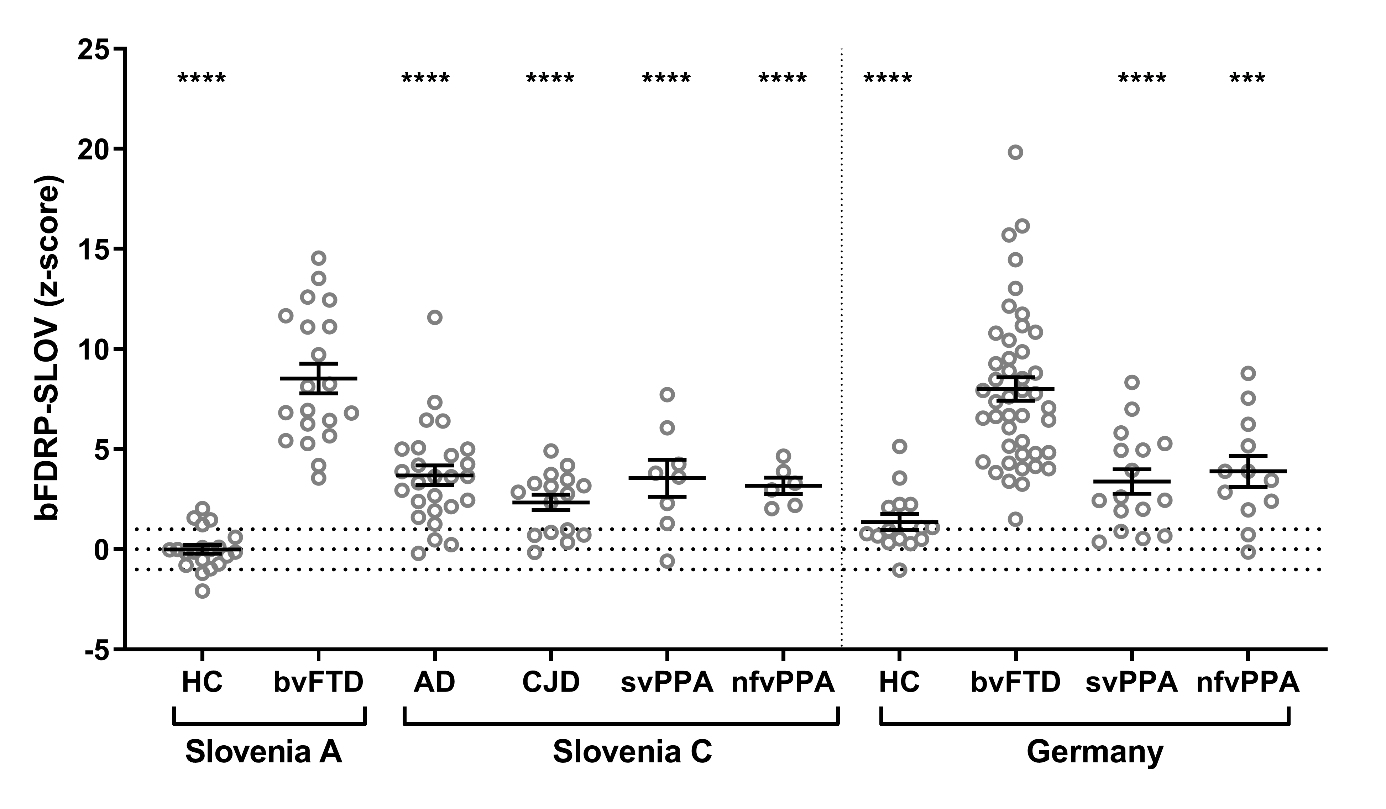


**Figure S3. bFDRP-SLOV expression scores of subjects from the cohorts Slovenia A, C, and Germany.**

Mean values and standard errors are presented. Vertical lines separates cohorts from different centers. One-way ANOVA was performed for each center independently. Subject scores differed in Slovenian (F_5,90_ = 29.4, p < 0.0001) and German subject groups (F3,83 = 20.8, p < 0.0001). Post-hoc Bonferroni’s multiple comparison test between bvFTD group and other groups are presented for both sites. NC – normal control subjects, bvFTD – behavioral variant frontotemporal dementia, AD – Alzheimer’s disease, CJD – sporadic Creutzfeldt-Jakob’s disease, svPPA – semantic variant primary progressive aphasia, nfvPPA – nonfluent variant primary progressive aphasia.


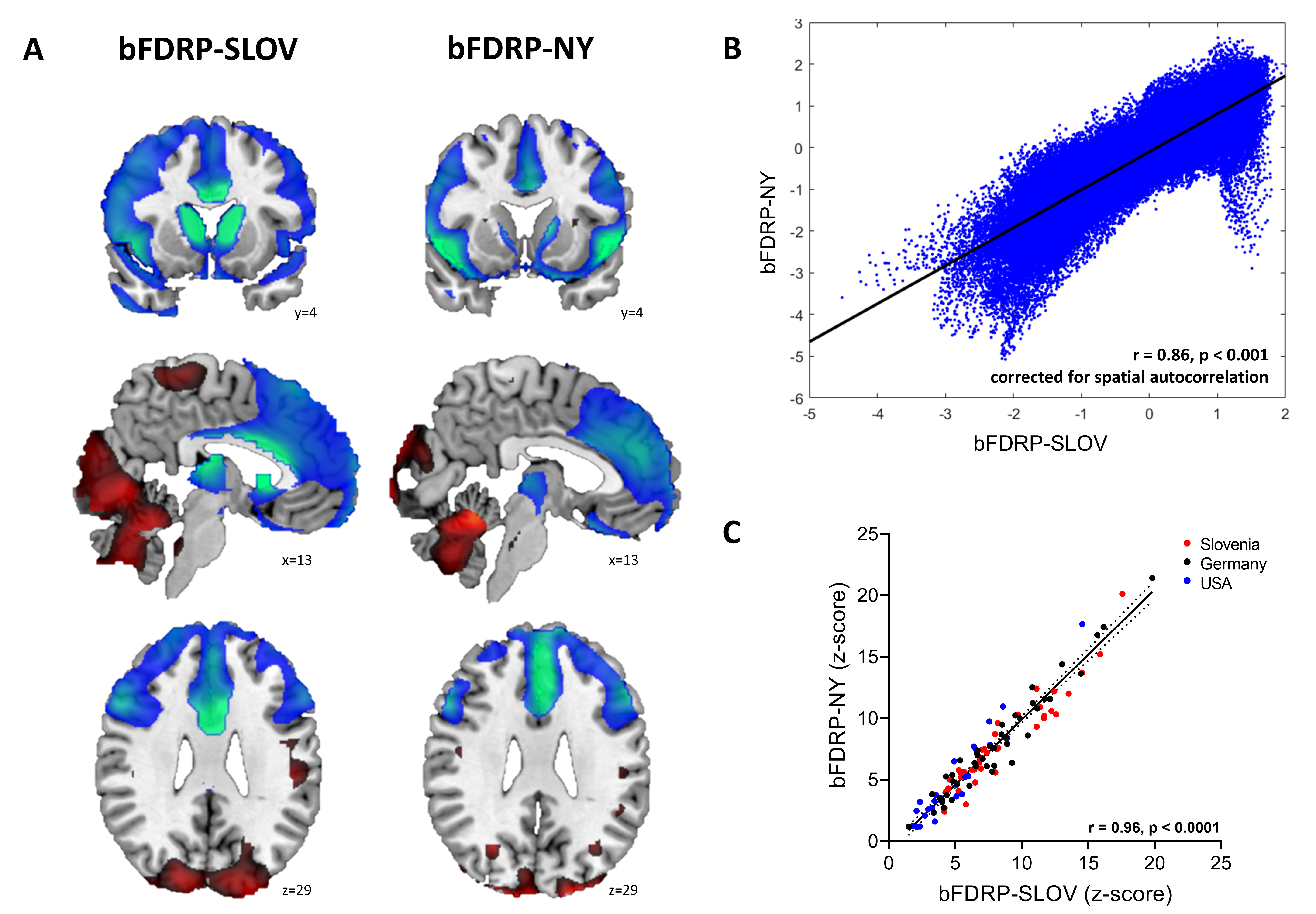


**Figure S4. bFDRP-NY derived from cohort USA subjects in comparison with the bFDRP-SLOV.**

(A) Topography of both bFDRP patterns. Relative hyperactive regions are color-coded red and relatively hypoactive regions color-coded blue. Coordinates in the axial (z), coronal (y) and sagittal (x) planes are in Montreal Neurological Institute (MNI) standard space. (B) - Significant correlation was found between bFDRP-SLOV and bFDRP-NY topographies (r = 0.86, p < 0.001, corrected for spatial autocorrelation) and (C) subject scores derived from both patterns (r = 0.96, p < 0.0001).


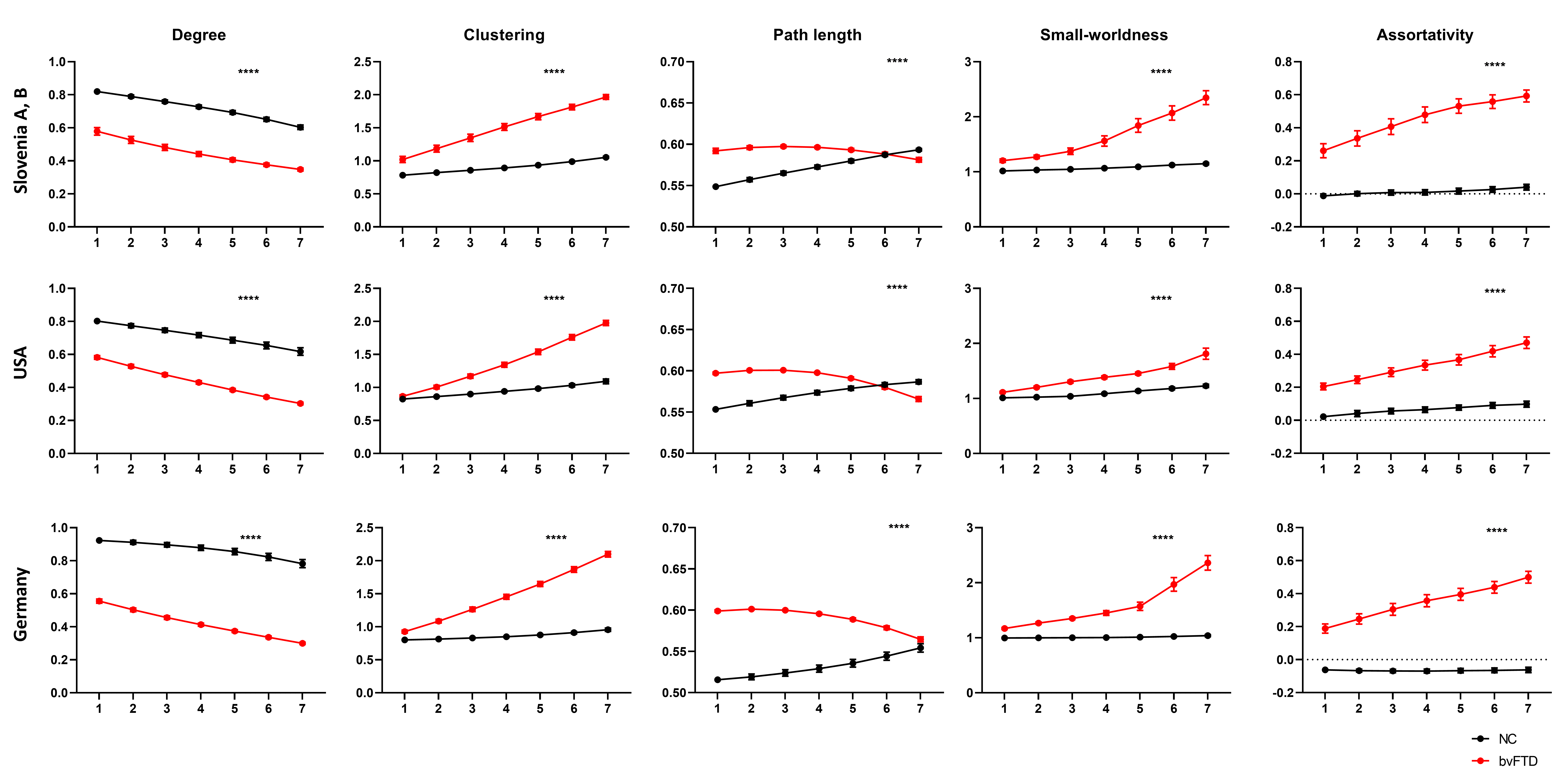


**Figure S5. Network parameters for the bFDRP-SLOV space in bvFTD patients and NC from all the three sites.**

In the bFDRP-SLOV vector space (n = 31 nodes) behavioral variant frontotemporal dementia (bvFTD) patients (red) showed (A) lower degree centrality and (B) higher clustering, (C) characteristic path length, (D) small-worldness and (E) assortativity than normal controls (NC) (black). [Threshold levels 1 to 7 correspond to cutoff thresholds r=0.3 to 0.6 in increments of 0.05. Two-way ANOVA test was used to determinate the difference between CJD and NC curves. **** - p < 0.0001]


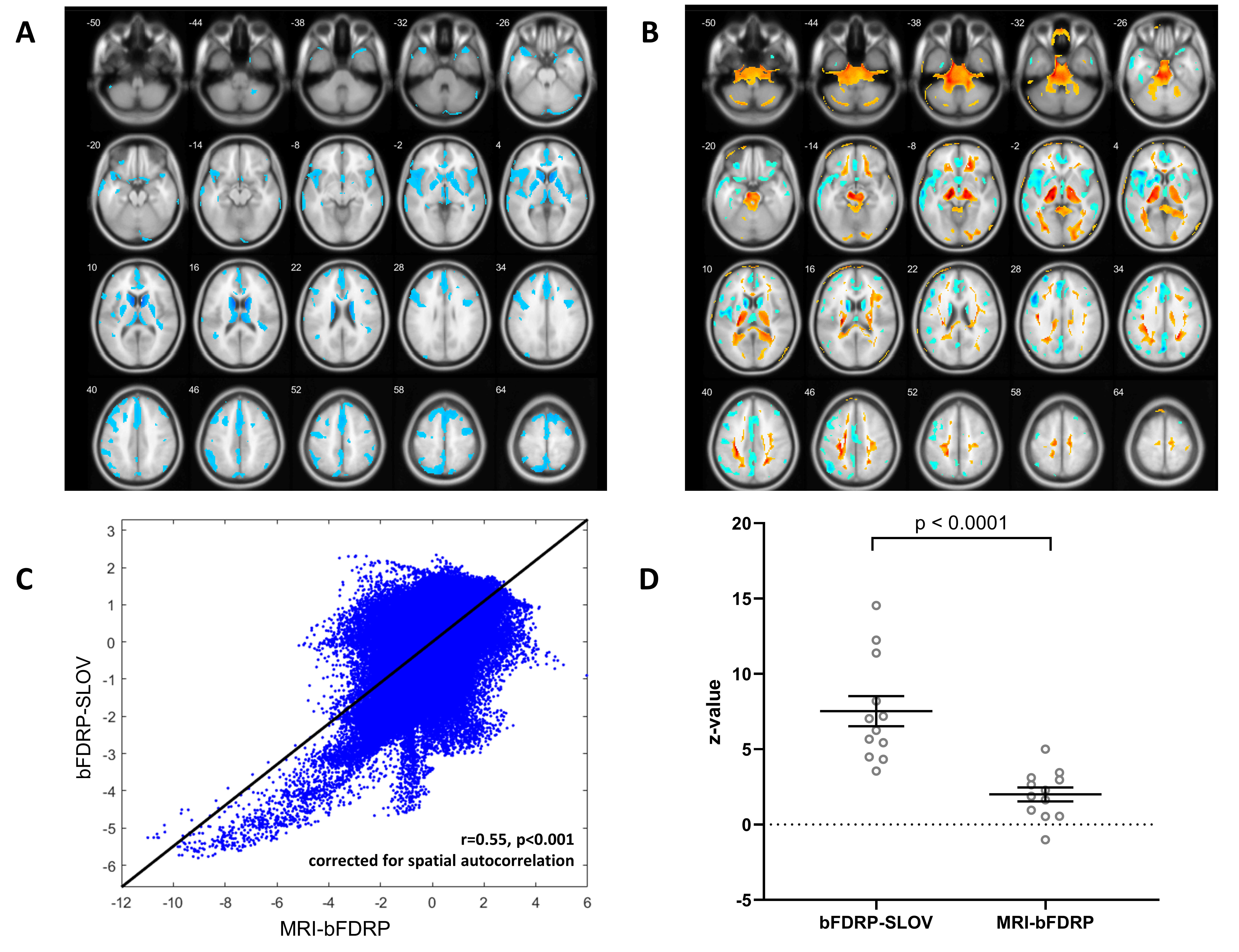


**Figure S6. Atrophy related MRI-bFDRP pattern.**

(A) Topography of the atrophy related MRI-bFDRP pattern was identified in a subset of 12 cohort Slovenia A and B patients and age-matched normal controls (NC) (see the Methods). (B) Bootstrap ICV map showing stability of the MRI-bFDRP pattern (p < 0.05; one sided, 1000 iterations) (atrophy is color-coded blue and relative structural preservation color-coded yellow to red). (C) The topography of atrophy related pattern MRI-bFDRP significantly correlated with the metabolic bFDRP pattern (r=0.55, p<0.001, corrected for spatial autocorrelation). (D) In the 12 bvFTD patients that underwent both FDG-PET and high resolution MRI, the corresponding z-scored values were considerably more expressed in the metabolic bFDRP-SLOV compared to the structural MRI-bFDRP pattern (p < 0.0001, paired *t*-test).


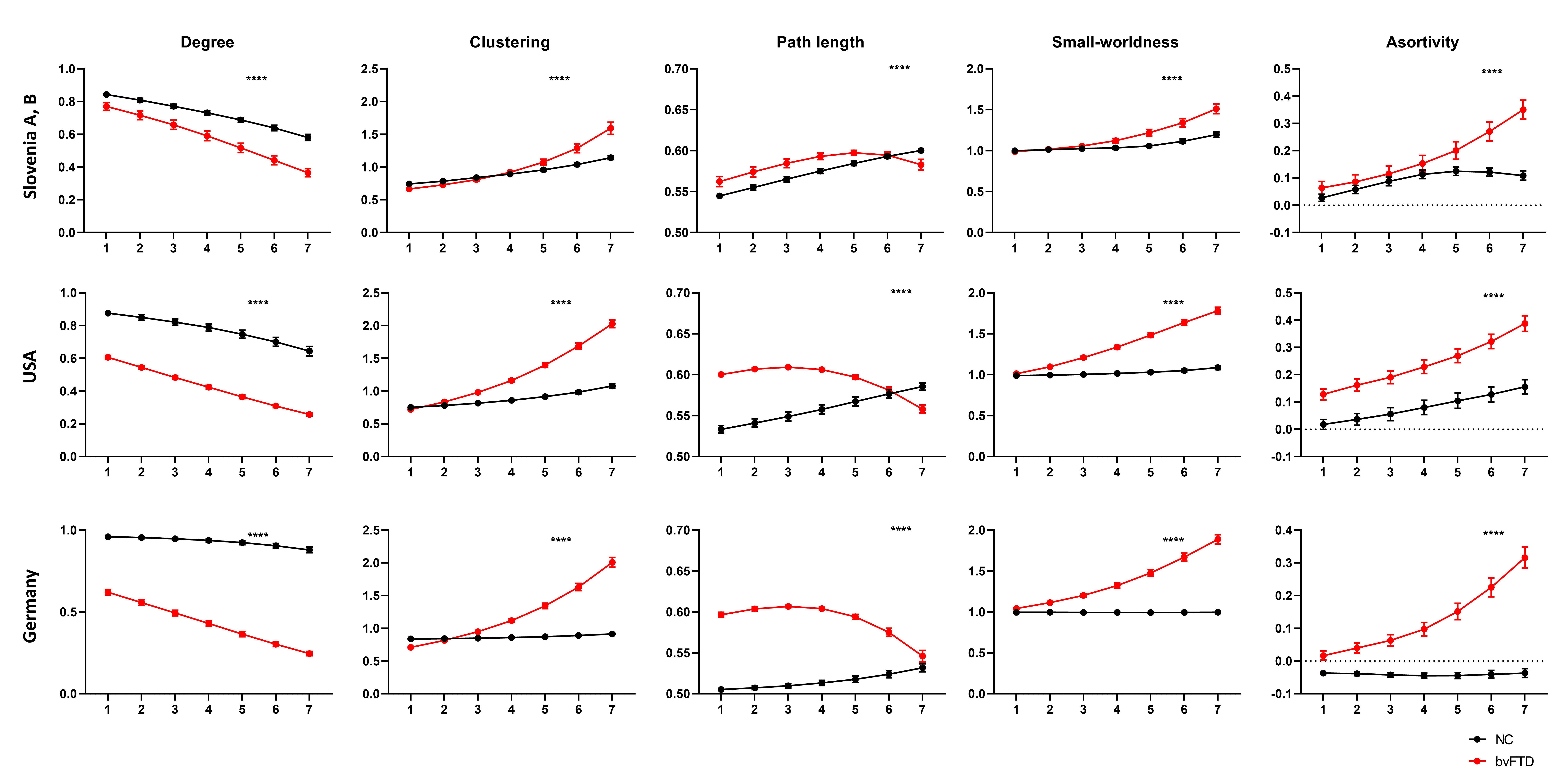


**Figure S7. Network parameters for the DMN space in bvFTD patients and NC from all the three sites.**

In the DMN vector space (n = 39 nodes) behavioral variant frontotemporal dementia (bvFTD) patients (red) just like in the bFDRP-SLOV space (Figure S6) showed (A) lower degree centrality and (B) higher clustering, (C) characteristic path length, (D) small-worldness and (E) assortativity than normal controls (NC) (black). [Threshold levels 1 to 7 correspond to cutoff thresholds r=0.3 to 0.6 in increments of 0.05. Two-way ANOVA test was used to determinate the difference between CJD and NC curves. **** - p < 0.0001]

**Supplementary Tables**

**Table S1.** **Brain regions with significant contribution to the bFDRP.** Clusters bigger than 70 voxels with intensity z > 1.64 (p < 0.05, one-tailed) are presented. Anatomic regions were identified according to the Automated Anatomical Labeling (AAL) atlas. Only relative hypometabolic regions are presented as none of the relative hyperactive z-transformed regions exceeded the cutoff p < 0.05.

| **Region (AAL3)** | **Voxels size** |
| --- | --- |
| ACC_pre_L | 114 |
| ACC_pre_R | 114 |
| ACC_sup_L | 488 |
| ACC_sup_R | 348 |
| Caudate_L | 644 |
| Caudate_R | 638 |
| Cingulate_Mid_L | 212 |
| Cingulate_Mid_R | 430 |
| Frontal_Inf_Oper_L | 339 |
| Frontal_Inf_Oper_R | 73 |
| Frontal_Inf_Orb_2_L | 347 |
| Frontal_Inf_Orb_2_R | 74 |
| Frontal_Inf_Tri_L | 649 |
| Frontal_Inf_Tri_R | 80 |
| Frontal_Mid_2_L | 881 |
| Frontal_Mid_2_R | 78 |
| Frontal_Sup_2_L | 859 |
| Frontal_Sup_Medial_L | 940 |
| Frontal_Sup_Medial_R | 476 |
| Insula_L | 350 |
| Insula_R | 116 |
| OFCant_L | 98 |
| OFClat_L | 137 |
| OFCpost_L | 77 |
| Precentral_L | 72 |
| Supp_Motor_Area_L | 210 |
| Supp_Motor_Area_R | 129 |
| Temporal_Pole_Sup_L | 277 |
| Temporal_Sup_L | 91 |
| Thal_MDm_L | 76 |
| Thal_PuM_R | 89 |

**Table S2.** **Brain regions with significant contribution to the disease progression related bvFTD-OrT/CVA pattern.** Clusters bigger than 70 voxels with intensity z > 1.64 (p < 0.05, one-tailed) are presented. Anatomic regions were identified according to the Automated Anatomical Labeling (AAL) atlas. Only relative hypometabolic regions are presented.

| **Region (AAL3)** | **Voxel size** |
| --- | --- |
| Caudate_L | 254 |
| Caudate_R | 289 |
| Frontal_Inf_Oper_L | 127 |
| Frontal_Inf_Oper_R | 166 |
| Frontal_Inf_Orb_2_L | 129 |
| Frontal_Inf_Tri_L | 213 |
| Frontal_Inf_Tri_L | 153 |
| Frontal_Med_Orb_L | 86 |
| Frontal_Med_Orb_R | 98 |
| Frontal_Mid_2_L | 163 |
| Frontal_Sup_2_L | 141 |
| Frontal_Sup_2_R | 136 |
| Frontal_Sup_Medial_L | 151 |
| Insula_L | 205 |
| Insula_R | 88 |
| Postcentral_L | 174 |
| Temporal_Sup_L | 78 |
| Thal_MDm_L | 86 |
| Thal_MDm_R | 93 |
